# Composites of Montmorillonite and Titania Nanoparticles Prepared by Inverse Microemulsion Method: Physico-Chemical Characterization

**DOI:** 10.3390/nano13040686

**Published:** 2023-02-10

**Authors:** Alicja Michalik, Bogna D. Napruszewska, Dorota Duraczyńska, Anna Walczyk, Ewa M. Serwicka

**Affiliations:** Jerzy Haber Institute of Catalysis and Surface Chemistry, Polish Academy of Sciences, Niezapominajek 8, 30-239 Krakow, Poland

**Keywords:** TiO_2_, clay, montmorillonite, organoclay, composite, inverse microemulsion, inverse micelle, mesoporosity

## Abstract

TiO_2_/montmorillonite composites were synthesized using inverse micellar route for the preparation of titania nanoparticles (4–6 nm diameter) in 1-hexanol and for the dispersion of one of the clay components. Two series of composites were obtained: one derived from cetyltrimethylammonium organomontmorillonite (CTA-Mt), exfoliated in 1-hexanol, and the other from sodium form of montmorillonite (Na-Mt) dispersed by formation of an inverse microemulsion in 1-hexanol. The TiO_2_ content ranged from 16 to 64 wt.%. The composites were characterized with X-ray diffraction, scanning/transmission electron microscopy/energy dispersive X-ray spectroscopy, thermal analysis, and N_2_ adsorption-desorption isotherms. The Na-Mt-derived component was shown to undergo transformation to CTA-Mt, as indicated by basal spacing of 17.5 nm, due to the interaction with the CTABr surfactant in inverse microemulsion. It was also better dispersed and intermixed with TiO_2_ nanoparticles. As a result, the TiO_2_/Na-Mt series displayed superior textural properties, with specific surface area up to 256 m^2^g^−1^ and pore volume up to 0.247 cm^3^g^−1^ compared with 208 m^2^g^−1^ and 0.231 cm^3^g^−1^, respectively, for the TiO_2_/CTA-Mt counterpart. Members of both series were uniformly mesoporous, with the dominant pore size around 5 nm, i.e., comparable with the dimensions of titania nanoparticles. The advantage of the adopted synthesis method is discussed in the context of other preparative procedures used for manufacturing of titania-clay composites.

## 1. Introduction

Titania-based composites are widely researched materials whose potential in heterogeneous catalysis and especially photocatalysis has long been recognized [1]. In catalysis, the TiO_2_ component is generally used as a catalyst carrier because it displays an advantageous combination of chemical inertness, resistance to poisoning, and the ability to interact with the supported active phase. In photocatalysis, titania plays the role of the actual catalyst, which, when subjected to bandgap illumination, enables generation of holes and electrons participating in the photochemical reaction.

The use of titania in the form of nanoparticles is particularly attractive because it ensures a high surface-to-volume ratio and high density of coordinatively unsaturated sites. This in turn affects the oxide stoichiometry, structure, electronic characteristics, and related properties such as, e.g., acido-basicity [2]. However, the use of nanoscale particles is burdened with the general challenges associated with their handling. The potential problems include the trickiness of nanoparticles separation and recovery from the reaction medium, the tendency to cluster at higher concentrations, or proneness to sintering at high temperatures [3,4]. These difficulties can be defused by anchoring the nanoparticles onto supports, the approach widely used in the design of titania-based systems. A great number of different inorganic and organic matrices have been used in the capacity of supports for TiO_2_ species. The examples include silica, zeolites, glass, clay minerals, carbonaceous materials, or organic polymers [5]. The naturally occurring clay minerals are of particular interest because they display an exceptional combination of advantageous properties, i.e., non-toxicity, abundance, low cost, and unique layered structures. The lamellar lattice elements may be considered as prefabricated building blocks ready to be used in advanced materials syntheses [6]. In addition, clay mineral surfaces are capable of physi- and/or chemisorption, which may be beneficial for enhancement of reagents concentration around the supported nanoparticles [7]. Several recent reviews reflect the widespread interest in various aspects of titania-clay composites synthesis and applications [1,8,9,10,11,12].

Montmorillonite is the mineral most frequently used in the capacity of clay component. Its cation exchange capacity and swellability are key properties controlling formation of the composites, and a variety of preparative approaches have been adopted in the past to design hybrids with TiO_2_ [1]. The most often employed procedure is referred to as pillaring. It results in the localization of polymeric Ti species in the interlayer, thereby increasing the layer spacing while maintaining the stacking order of the layers. Another type of nanoarchitecture is formed when TiO_2_ nanoparticles become trapped between disordered, exfoliated montmorillonite layers. This type of structure is called a house of cards assembly. Alternatively, when the synthesis conditions prevent cation exchange and/or swelling, the titania nanoparticles become attached to the external surfaces of the mineral grains. This type of TiO_2_ deposition is usually the case during montmorillonite impregnation with the Ti precursor but may also appear in addition to the interlayer loading.

The common method used for generation of titania species to be embedded in the composite structure is the in situ or ex situ hydrolysis of inorganic or organic precursor [1]. Recently, we described a novel strategy for design of metal oxide/clay composites based on the use of exfoliated layered organoclays but with oxide nanoparticles obtained from oxohydroxy precursors prepared in water-in-oil (w/o) inverse microemulsions [13,14,15]. Inverse microemulsions, also referred to as reverse microemulsions, are thermodynamically stable and optically transparent dispersions of aqueous microdroplets in a continuous oil phase and stabilized by the presence of surfactant molecules at the water–oil interface [16]. In the ternary water-oil-surfactant phase diagram, the region in which the inverse micelles are formed is usually found near the oil apex of the triangle [17]. Mixing of inverse microemulsions containing appropriate reactants in aqueous micellar cores results in coalescence of micelles and precipitation of nanoparticles, with size limited by the dimensions of micellar interior. Moreover, the variation of the water-to-oil ratio and surfactant concentration could influence the size of prepared nanoparticles. For this reason, the inverse microemulsion method has been widely used for the synthesis of variety of nanomaterials [16,17,18,19].

In the present work, we describe the application of the inverse micellar route to produce titania nanoparticles for the preparation of TiO_2_/clay composites. The clay component, montmorillonite (Mt), was prepared in two different ways. In the first case, Mt was used as a cetyltrimethylammonium organoclay (CTA-Mt) to facilitate its exfoliation in the organic reaction medium (1-hexanol). In the other procedure, Na-montmorillonite (Na-Mt) was used. This form of clay mineral is hydrophilic and does not disperse in n-hexanol. To bypass this obstacle, we used a completely novel approach in which the Na-Mt component was prepared as an inverse microemulsion in 1-hexanol, with clay mineral present as a suspension in aqueous micellar cores.

## 2. Materials and Methods

### 2.1. Materials

The starting montmorillonite (Mt) used for the preparation of composites was the sodium form of the less than 2 µm particle size fraction separated by sedimentation from Jelšový Potok (JP) bentonite (Slovakia) provided by ZGM Zebiec (Starachowice, Poland). The JP deposit contains montmorillonite as the only clay mineral phase [20].

The ternary system water/cetyltrimethylammonium bromide (CTA-Br)/1-hexanol with well-known phase diagram [21] was chosen for the preparation of microemulsions. Based on this diagram, the weight proportion of aqueous phase:CTABr:1-hexanol equal 17:28:55 was selected for the preparation of inverse microemulsions. The mixture is characterized by the molar water-to-surfactant ratio w_0_ = 11.75. Parameter α (the ratio of oil phase mass to the sum of oil and aqueous phase masses) is =0.76, and parameter γ (the ratio of surfactant mass to the sum of oil, water, and surfactant masses) is 0.28.

The following reactants, denoted as A, B, C, and D, were used in the composite syntheses:

A. The sodium form of montmorillonite, denoted as Na-Mt, was obtained by cation exchange of parent clay with excess of 1 M NaCl solution, washed by centrifugation with distilled water till negative reaction of the solute with AgNO_3_, and stored in the form of a gelatinous paste (ca. 5 wt.% of clay);

B. The organomontmorillonite was prepared from Na-Mt by cation exchange with excess of cetyltrimethylammonium bromide (CTABr) aqueous solution, followed by washing with distilled water till negative reaction of the solute with AgNO_3_. To remove excess water, the organic derivative, denoted as CTA-Mt, was washed three times with isopropanol, then three times with 1-hexanol, and stored in the form of a gelatinous paste (ca. 10 wt.% of clay);

C. The inverse Ti-containing microemulsion was prepared by adapting the approach proposed for the microemulsion synthesis of hydrous zirconia nanoparticles [22]. First, the Ti-containing aqueous phase was obtained by controlled hydrolysis of TiCl_4_ [23]. In a typical experiment, 6.5 mL of 6 M HCl was added to 9 mL of TiCl_4_ and diluted with 100 mL of distilled water. After stirring for 3 h at room temperature, a clear solution containing 3.7 wt.% of Ti was obtained. The Ti aqueous phase was mixed with CTABr and 1-hexanol in weight proportion 17:28:55 and stirred till the mixture turned into a dense transparent liquid, indicating formation of inverse microemulsion;

D. The microemulsion of NH_3_aq, used for speeding up hydrolysis of the Ti precursor species, was obtained by mixing 25% NH_3_aq, CTABr, and 1-hexanol in the selected weight proportion till the formation of clear solution.

TiCl_4_ (ReagentPlus^®^), CTABr (≥98%), and 1-hexanol (for synthesis) were purchased from Sigma-Aldrich (Poznan, Poland); all other chemicals used in syntheses were of p.a. purity provided by Chempur (Piekary Slaskie, Poland). 

**Figure 1:**
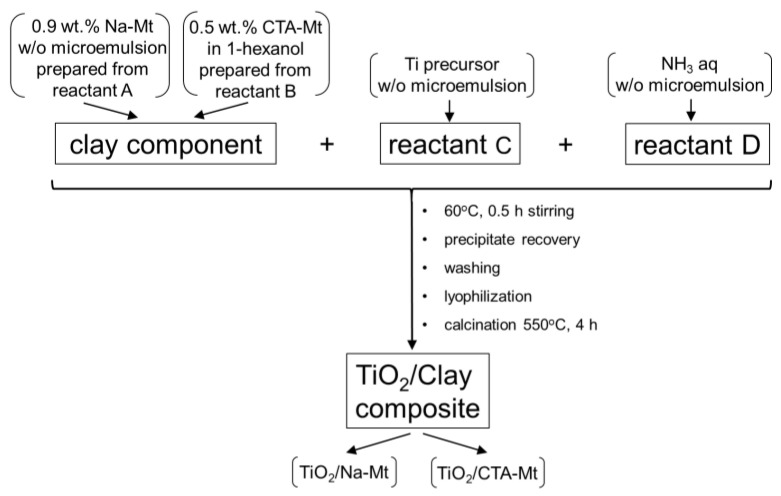
Schematic illustration of composite synthesis.

Two methods of composite synthesis, differing by the manner of preparation of the clay mineral component, were used. Organomontmorillonite to be used in composite synthesis was prepared by dispersing reactant B in 1-hexanol to yield 0.5 wt.% suspension of CTA-Mt. Sodium montmorillonite was prepared as a microemulsion of Na-Mt by mixing reactant A with CTABr and 1-hexanol in weight proportion 17:28:55 and stirring the mixture till a clear, gray liquid containing ca. 0.9 wt.% Na-mt was obtained. Further synthesis stages were common for both the CTA-Mt and the Na-Mt components. The clay mineral dispersions were mixed with reactant C added in the amount providing the intended TiO_2_ loading of 0.25, 0.5, 1, or 1.5 g per gram of montmorillonite component and heated to 60 °C. To accelerate Ti precursor hydrolysis, reactant D was added under stirring till the originally greyish suspensions turned white. The mixtures were kept under stirring at 60 °C for 30 min. After cooling down, the suspensions were washed by centrifugation with ethanol (3×), followed by washing with 1:1 water-ethanol mixture until free of bromide ions. Lyophilization of the precipitates yielded fine powders that were subjected to calcination for 4 h at 550 °C. The samples obtained from CTA-Mt are referred to as 0.25TiO_2_/CTA-Mt, 0.5TiO_2_/CTA-Mt, 1TiO_2_/CTA-Mt, and 1.5TiO_2_/CTA-Mt, while those prepared from Na-Mt are denoted as 0.25TiO_2_/Na-Mt, 0.5TiO_2_/Na-Mt, 1TiO_2_/Na-Mt, and 1.5TiO_2_/Na-Mt. A scheme of composite synthesis is shown in Figure 1.

### 2.2. Methods

Powder X-ray diffraction (XRD) patterns were recorded using X’Pert PRO MPD diffractometer (PANalytical, Almelo, The Netherlands) with CuKα radiation. The crystallite sizes of TiO_2_ nanoparticles (the size of coherently scattering domains) were estimated for the (101) reflection of anatase using the Scherrer formula and taking into account the instrumental broadening. Scanning/transmission electron microscopy/energy dispersive X-ray spectroscopy (SEM/TEM/EDX) study of the materials was carried out with aid of JEOL JSM-7500F Field Emission Scanning Electron Microscope (JEOL, Tokyo, Japan) coupled with an INCA PentaFetx3 EDX (Oxford Instruments, Abingdon, UK) system. SEM/TEM images were recorded for the uncoated samples deposited as suspensions on 200 mesh copper grids covered with carbon support film. An average of EDX measurements for 10 areas of ca. 1 μm × 1 μm, chosen at random on the sample surface, was used for determination of sample composition. N_2_ adsorption/desorption at −196 °C was measured with an AUTOSORB 1 (Quantachrome, Boynton Beach, FL, USA) instrument. The samples were outgassed at 200 °C for 2 h. Brunauer–Emmett–Teller (BET) formalism was used for the calculation of specific surface areas (S_BET_). The total pore volume (V_tot_) was determined from the amount of N_2_ adsorbed at p/p_0_ = 0.996. Mesopore volume (V_meso_) was determined from the adsorption branch using the Barrett–Joyner–Halenda (BJH) method. Pore size distribution (PSD) profiles were determined from the adsorption branch using the non-local density functional theory (NL DFT) method. The mean pore diameter (D_av_) was calculated with the D_av_ = 4V_tot_/S_BET_ Gurvitch formula. The Specac mini pellet press (Specac Ltd., Orpington, UK) was used for pelletization of the composite powder. A combined thermogravimetric (TG) and differential scanning calorimetry (DSC) analysis was carried out in the flow of air (40 mL/min) with a STA 409 PC LUXX TG/DSC apparatus (Netzsch, Selb, Germany) in the temperature range of 30–1000 °C and at a heating rate of 10 °C/min.

## 3. Results and Discussion

### 3.1. Electron Microscopy Analysis

**Figure 2:**
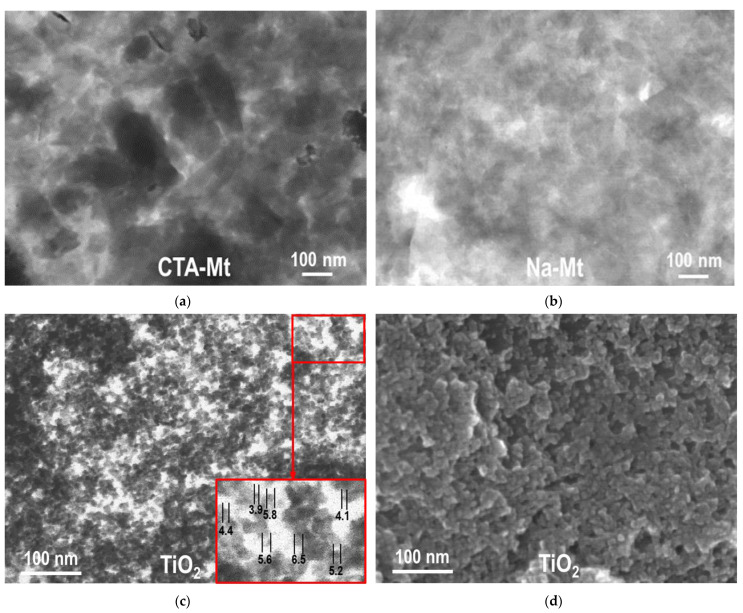
(**a**) TEM image of CTA-Mt; (**b**) TEM image of Na-Mt; (**c**) TEM image of titania obtained by inverse microemulsion method; (**d**) SEM image of titania obtained by inverse microemulsion method.

TEM and SEM micrographs of individual components used for the synthesis of composites are gathered in Figure 2. Figure 2a,b show TEM images of dried suspensions of CTA-Mt and Na-Mt, respectively. Both Mt dispersions are composed of very fine platelets. On average, the particles present in Na-Mt are thinner and smaller than those found in organoclay, pointing to the better dispersion of clay mineral suspension prepared by inverse microemulsion method. Figure 2c,d show a TEM and SEM image of TiO_2_ nanoparticles prepared by inverse microemulsion. The material is composed of very fine, rounded grains of ca. 4–6 nm diameter (blown up images in Figure 2c).

**Figure 3:**
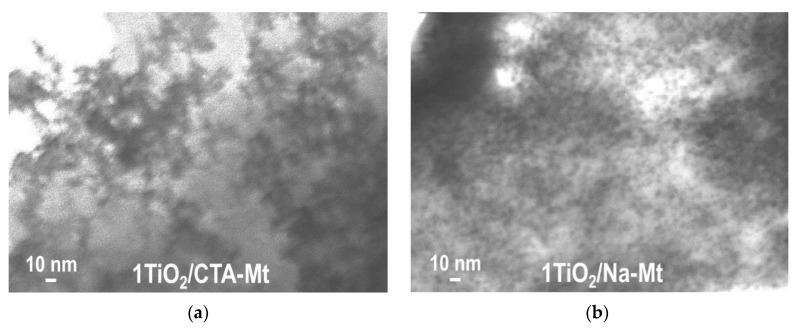
TEM images of composites: (**a**) 1TiO_2_/CTA-Mt; (**b**) 1TiO_2_/Na-Mt.

TEM analysis revealed differences between the TiO_2_/CTA-Mt and TiO_2_/Na-Mt series in the degree of intermixing of the two components. The effect observed for all members of the series is illustrated in Figure 3 with images of 1TiO_2_/CTA-Mt and 1TiO_2_/Na-Mt samples. In the micrographs, titania is visible as dark particles embedded in the clay mineral matrix. In 1TiO_2_/Na-Mt (Figure 3b), the titania nanoparticles are clearly better dispersed and quite homogeneously distributed. On the other hand, in 1TiO_2_/CTA-Mt (Figure 3a), agglomerates of TiO_2_ species are more frequent, and the coverage of clay matrix by TiO_2_ is less even. Results of the SEM/TEM study of individual components (Figure 2) suggest that the better intermixing of components in the TiO_2_/Na-Mt series is likely to be due to the better dispersion/disintegration of the Na-Mt component. The reason for the observed effect may be differences in the preparation of suspensions of clay components. It is known that disintegration of Mt particles in the solvent is prompted by the swelling of clay mineral due to the penetration of solvent molecules into the interlayer. The resulting expansion of the interlayer spacing facilitates disassociation/exfoliation of clay particles. Swelling and exfoliation occurs both in the case of CTA-Mt dispersed in a suitable organic solvent and in the case of Na-Mt dispersed in water, but the phenomenon is much more efficient in the latter case [24]. Moreover, in contrast to the conventional dispersion of CTA-Mt in 1-hexanol, in the Na-Mt microemulsion, the nanoparticles of exfoliated clay are held within the aqueous micellar cores and are, therefore, additionally stabilized against restacking and reaggregation.

**Figure 4:**
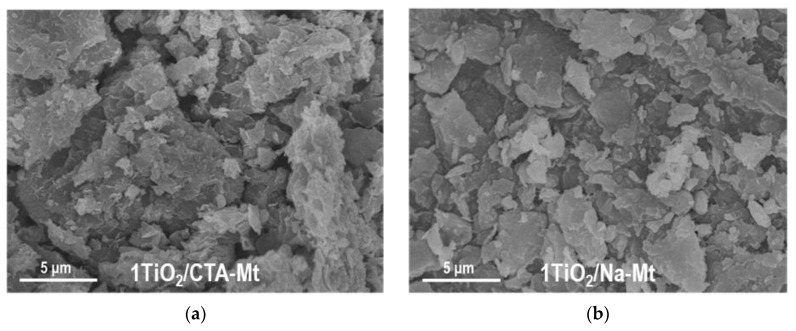

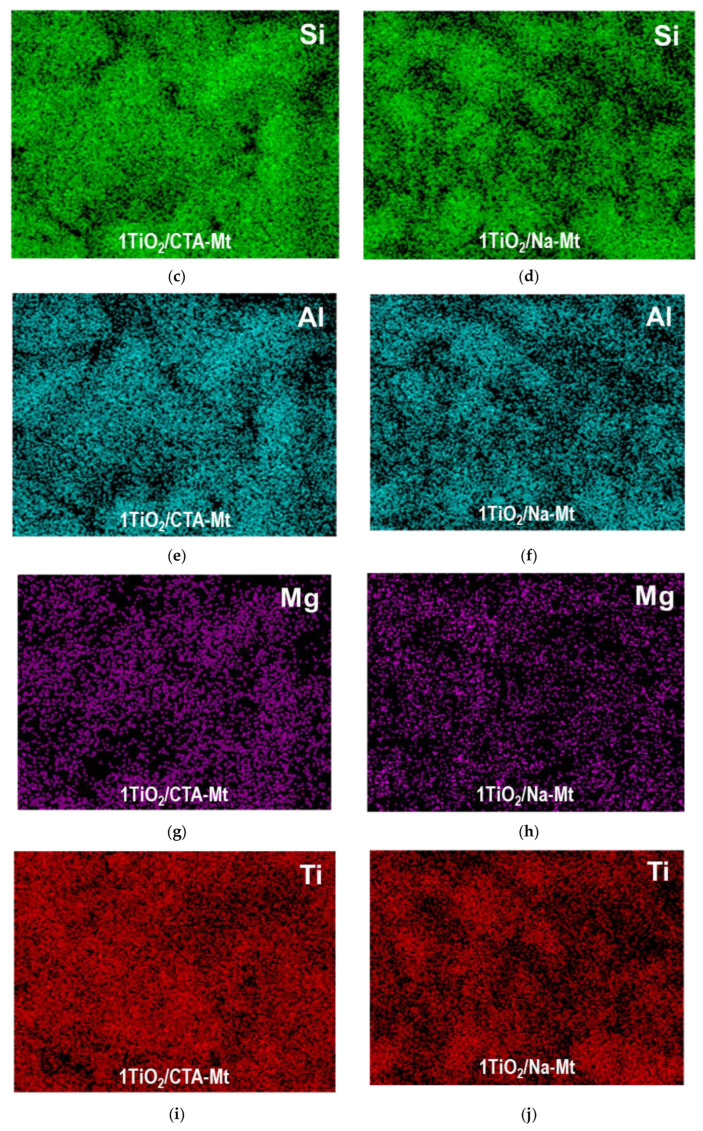
SEM/EDX compositional analysis of selected areas of 1TiO_2_/CTA-Mt and 1TiO_2_/Na-Mt composites: (**a**) SEM image of 1TiO_2_/CTA-Mt; (**b**) SEM image of 1TiO_2_/Na-Mt; (**c**) EDX mapping image of Si in 1TiO_2_/CTA-Mt; (**d**) EDX mapping image of Si in 1TiO_2_/Na-Mt; (**e**) EDX mapping image of Al in 1TiO_2_/CTA-Mt; (**f**) EDX mapping image of Al in 1TiO_2_/Na-Mt; (**g**) EDX mapping image of Mg in 1TiO_2_/CTA-Mt; (**h**) EDX mapping image of Mg in 1TiO_2_/Na-Mt; (**i**) EDX mapping image of Ti in 1TiO_2_/CTA-Mt; (**j**) EDX mapping image of Ti in 1TiO_2_/Na-Mt.

The less homogeneous titania dispersion in the TiO_2_/CTA-Mt series, compared to the TiO_2_/CTA-Mt series, is also observed at the macroscale, as illustrated by SEM/EDX mapping of titanium and key clay structure-forming elements, i.e., Si, Al, and Mg, in 1TiO_2_/CTA-Mt and 1TiO_2_/Na-Mt composites. The results are shown in Figure 4. For a given composite, the mappings of Si, Al, and Mg mirror each other and reflect the texture/morphology of clay particles visible in SEM images. As for the distribution of Ti, it is evident that, in both samples, it is spread throughout the analyzed material, but only in 1TiO_2_/Na-Mt does the mapping of Ti replicate the sample texture/morphology in a similar way as the maps of clay-forming elements, pointing to a homogeneous intermixing of titania with the clay matrix. In 1TiO_2_/CTA-Mt, the match between the map of Ti and the maps of Si, Al, and Mg is much poorer, which indicates less even blending of titania nanoparticles with clay component.

### 3.2. X-ray Diffraction Analysis

**Figure 5:**
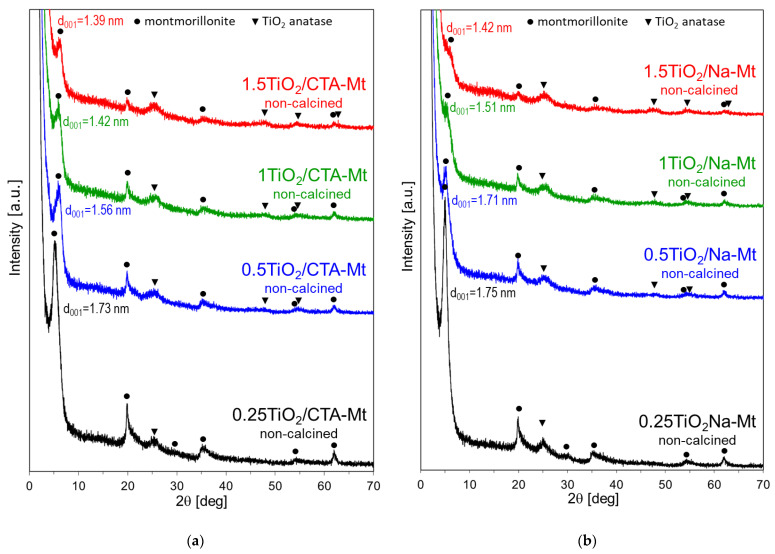
XRD patterns of non-calcined composites: (**a**) TiO_2_/CTA-Mt and (**b**) TiO_2_/Na-Mt; d_001_—basal spacing of Mt component.

**Figure 6:**
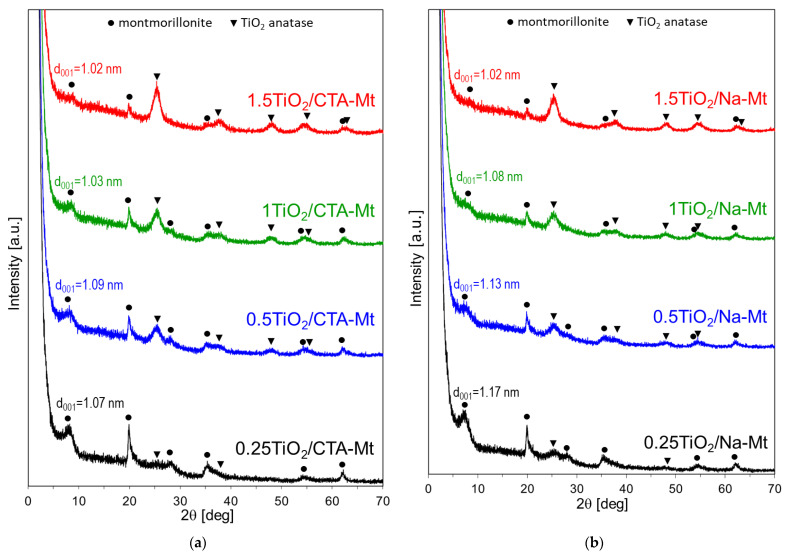
XRD patterns of calcined composites: (**a**) TiO_2_/CTA-Mt and (**b**) TiO_2_/Na-Mt; d_001_—basal spacing of Mt component.

XRD patterns of the investigated composite materials before and after calcination are shown in Figure 5 and Figure 6, respectively. In all cases, reflections due to both composite components are visible. Thus, in the case of the non-calcined TiO_2_/CTA-Mt series, montmorillonite reflections are observed, the intensity of which decreases with growing titania loading, as well as reflections assignable to poorly crystalline anatase modification, the intensity of which increases with Ti content. The result shows that hydrolysis of Ti precursor leads to the formation of some poorly crystalline titania already prior to calcination. The non-calcined 0.25TiO_2_/CTA-Mt sample displays only the most intense (101) reflection of anatase at 2Θ = 25.3°, but as the Ti loading increases, other anatase reflections become distinguishable (Figure 5a). In non-calcined 0.25TiO_2_/CTA-Mt, the d_001_ basal spacing of Mt component is 1.73 nm, which is consistent with the bilayer conformation of aliphatic chains of organocation in the interlayer [25]. As TiO_2_ loading grows, the d_001_ spacing of Mt becomes gradually lower to assume 1.39 nm in non-calcined 1.5TiO_2_/CTA-Mt, which points to a monolayer arrangement of alkylammonium cations. The shift is attributed to the partial exchange of interlayer CTA^+^ cations with hydronium ions stemming from the acidic aqueous cores of Ti inverse micelles. The lowering of CTA^+^ content in the interlayer enables single-layer packing of organocations. Interestingly, the XRD patterns of non-calcined TiO_2_/Na-Mt series show that instead of the basal spacing expected for sodium form of Mt (ca. 1.2 nm), the Mt component displays larger d_001_, which is characteristic of an organoclay (Figure 5b). It is apparent that during preparation of Na-Mt dispersion by means of inverse micellar method, the CTABr surfactant acts as a source of organocations, leading to exchange of interlayer Na^+^ with CTA^+^. This conclusion is supported by the results of EDX experiments, which find no sodium in the TiO_2_/Na-Mt composites (Table 1). The effect is advantageous, as in certain catalytic and photocatalytic applications, the presence of sodium impurity is detrimental [13,26]. As in the non-calcined TiO_2_/CTA-Mt samples, a shift in the basal spacing from 1.75 to 1.42 nm is also observed in the TiO_2_/Na-Mt composites, reflecting the gradual exchange of CTA^+^ with hydronium ions.

Calcination of composites brings about two major changes in the XRD patterns of both series (Figure 6). First, the clay components display basal spacing d_001_ equal ca. 1 nm, characteristic of thermally collapsed Mt, and second, the reflections of anatase become more intense, indicating further crystallization of TiO_2_ nanoparticles. The size of titania crystals, estimated by means of the Scherrer equation, is about 5–5.5 nm in both types of composites, which is comparable with the size of TiO_2_ nanoparticles detected by electron microscopy.

### 3.3. Thermal Analysis

**Figure 7:**
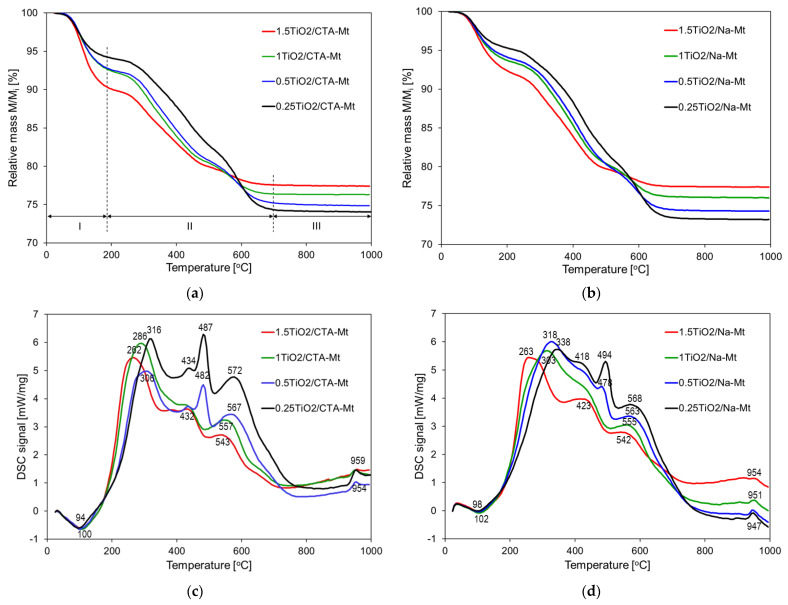
Thermal analysis of non-calcined composites: (**a**) TG of TiO_2_/CTA-Mt samples; (**b**) TG of TiO_2_/Na-Mt samples; (**c**) DSC of TiO_2_/CTA-Mt samples; (**d**) DSC of TiO_2_/Na-Mt.

Thermal evolution of non-calcined composites was studied by means of combined TG/DSC analysis, and the results are presented in Figure 7. Both series of composites show TG traces of similar shapes, and the total weight losses for the samples of analogous TiO_2_ loadings are comparable (Figure 7a,b). This observation confirms that Na-Mt component underwent transformation to CTA-exchanged form during formation of microemulsion, so compositionally, both series of non-calcined composites are alike. Three stages of thermal decomposition can be distinguished in TG traces, as marked in Figure 7a. In the first, below 180 °C, mass loss increases with TiO_2_ loading, in the second, up to ca. 700 °C, the opposite trend is observed, i.e., the lower the TiO_2_ content, the higher the mass loss, and in the third, the mass of the samples stabilizes, with total mass loss growing with increasing content of Mt. The observed effects may be explained by considering concurrent thermal decomposition of composite components, i.e., the organoclay and the oxy-hydroxide Ti species. In the first stage of decomposition, below 180 °C, dehydration of both components occurs and is accompanied by the endothermic effect with maximum around 100 °C (Figure 7c,d). However, the organoclay component is relatively hydrophobic, and numerous studies have shown that only little water is desorbed in this temperature range [27,28,29]. Thus, in the composites, the initial mass loss is dominated by the dehydration of the hydrous titania precursor, and it therefore increases with the intended TiO_2_ loading. The second stage of mass loss is dominated by the oxidative decomposition of the surfactant molecules adsorbed at the Mt surface and present as interlayer CTA^+^ cations. The process occurs in several overlapping steps accompanied by strong exothermic effects, with the last maximum in the 540–570 °C range (Figure 7c,d) due to the combustion of charcoal deposit [30]. The mass loss due to the dehydroxylation of Mt layers, known to occur below 700 °C, cannot be distinguished from the principal mass loss caused by combustion of the organic matter. Additionally, the accompanying endothermic effect is obscured by the strong exothermic maxima related to the oxidation of the organic matter. It is noteworthy that in both series, the exothermic maximum marking the final combustion of the organic deposit shifts from ca. 570 to ca. 540 °C upon growing TiO_2_ content, pointing to the catalytic action of titania. In the third stage of thermal evolution, the mass of the samples remains constant, and the exothermic effect around 950 °C is due to the recrystallization of decomposed montmorillonite lattice; hence, its magnitude diminishes with the falling share of Mt component.

### 3.4. Textural Properties

**Figure 8:**
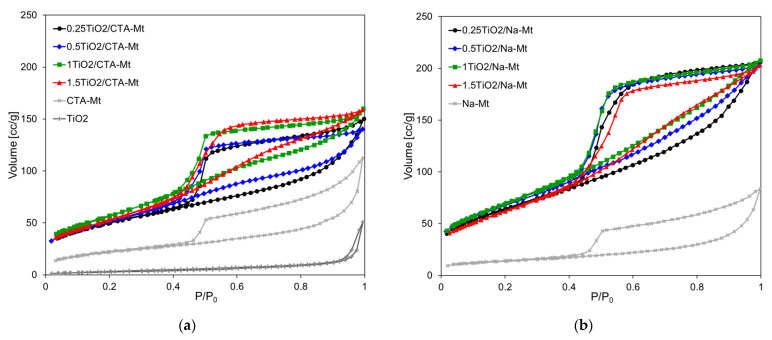

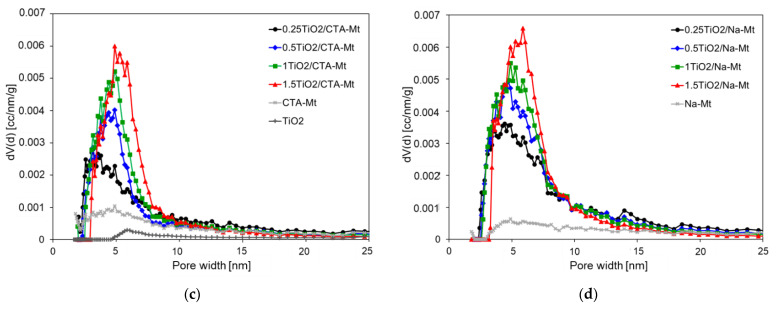
N_2_ adsorption/desorption isotherms at –196^o^C of: (**a**) TiO_2_/CTA-Mt; (**b**) TiO_2_/Na-Mt. Differential pore size distribution (dV(**d**)) of: (**c**) TiO_2_/CTA-Mt; (**d**) TiO_2_/Na-Mt.

The nitrogen adsorption/desorption isotherms of TiO_2_/CTA-Mt and TiO_2_/Na-Mt series, together with the data obtained for individual components subjected to the same calcination procedure as the composites, are shown in Figure 8a,b, respectively. Textural parameters calculated from these graphs are presented in Table 1.

The shapes of adsorption isotherms of both series are very similar and may be classified as Type II, following the IUPAC classification [31]. All composites display pronounced hysteresis loops of H3 type, according to the same classification. The characteristic feature of H3 loop is that it closes at about p/p_0_ = 0.42, which is typical of cavitation-induced pore emptying. Loops of this type are given by non-rigid aggregates of plate-like particles, which is consistent with the house of cards structure. The isotherms resemble those of individual clay components but are shifted upwards, pointing to the higher adsorption capacity of the composites. The isotherm of the calcined TiO_2_ component lies much lower and displays almost no hysteresis, which indicates low porosity and suggests the occurrence of sintering during thermal treatment. Besides similarities, there are also distinct differences between the TiO_2_/CTA-Mt and TiO_2_/Na-Mt series. The adsorption capacity of the latter is clearly higher, and larger hysteresis loops point to larger mesoporous volume. Indeed, the calculated values of the total pore volumes and the mesopore volumes gathered in Table 1 show that TiO_2_/Na-Mt samples are characterized by higher V_tot_ values than their TiO_2_/CTA-Mt counterparts and that in each case the difference results from the increase of V_meso_. It is noteworthy that the texture of the composites is clearly better developed than that of calcined parent clays. The pore size distribution curves presented in Figure 8c,d show that the dominant pore size, indicated by the PSD curve maximum, shifts slightly to higher values as TiO_2_ loading increases. The dominant pore sizes of samples with the lowest TiO_2_ content, namely 0.25TiO_2_/CTA-Mt and 0.25TiO_2_/Na-Mt, are about 3.8 and 4.5 nm and thus are meaningfully lower than the calculated average pore dimensions of 5.3 and 5.8 nm, respectively (Table 1). In all samples with higher TiO_2_ content, the values of the dominant pore size and the average pore dimension are pretty close, pointing to the uniform character of pore network in composites richer in titania component. It should be noted that the dominant pore dimensions, around 5 nm, are close to the size of TiO_2_ particles determined by TEM analysis (4–6 nm) and to the anatase crystallite dimension estimated from XRD (5–5.5 nm), which may be taken as an indication that the presence of uniform TiO_2_ grains formed within inverse micelles is the main factor shaping the mesoporosity of composites.

Textural properties of TiO_2_/clay composites are of particular importance for potential applications, as the accessibility of titania by the reactants depends on the well-developed pore network. Table 2 enables comparison of specific surface area and pore volume of selected examples of titania-clay composites obtained according to various synthetic procedures described in the literature, with the relevant properties of materials synthesized in this study. As mentioned in the Introduction, TiO_2_/clay composites prepared by pillaring are the most commonly studied. Synthesis variations are numerous and involve, among others, change of calcination temperature, the use of different pillaring agents, the application of hydrothermal treatment, the use of organoclay host, etc. It is known that although composites obtained by pillaring display high specific surface area and pore volume, their largely microporous character may result in reactants experiencing diffusional limitations [1,11,12,32]. Microporosity can be largely reduced during synthesis of titania-clay heterostructures, whereby the layered clay host becomes exfoliated [33]. Composites prepared by impregnation ensure good accessibility of titania but typically possess much lower specific surface and pore volume [34]. With respect to the value of specific surface area and the total pore volume, the samples synthesized in this work compare favorably with most of the presented examples. Moreover, the TiO_2_/CTA-Mt and TiO_2_/Na-Mt series, although well-developed texturally, are non-microporous and show uniform mesoporous profiles, which is the feature important in design of potential photocatalysts or catalysts. The 1TiO_2_/Na-Mt powder with the best-developed specific surface area and pore volume was tested for resistance of porous texture to mechanical stress by subjection to 1-ton pressure in a hydraulic press. The material proved quite robust, as its specific surface area remained almost unchanged (250 vs. 256 m^2^g^−1^ before compression), while the total pore volume diminished by ca. 20% (0.261 vs. 0.321 cm^3^g^−1^ before compression).

It should be recalled that the interest in the development of titania-based nanocomposites is not limited to photocatalysis and catalysis but also stems from their wide application in photovoltaics, hydrogen storage, sensors, biomedicine, and electronics [1,8,9,10,11,12,40,41,42,43,44,45,46]. The synthetic procedure described in this work is aimed specifically at the formation of titanium-clay hybrid systems but can be readily adapted to the deposition of TiO_2_ nanoparticles in a variety of matrices or can be used to prepare finely dispersed clay nanoparticles for potential use in other clay-based composites such as, e.g., clay-reinforced polymers. The proposed preparative approach can therefore inspire the design and fabrication of novel hybrid systems with potential well beyond the application areas of TiO_2_/clay composites [47].

## 4. Conclusions

The method of TiO_2_/clay composite synthesis proposed in this work bases on the use of microemulsion technique for the synthesis of TiO_2_ component and/or preparation of clay dispersion. The adopted procedure yielded uniform, rounded TiO_2_ particles of 4–6 nm diameter. The new approach enabled dispersion of hydrophilic Na-Mt in an organic medium by formation of inverse microemulsion with clay particles present in aqueous micellar cores. The procedure resulted in the Na-Mt transformation to CTA-exchanged Mt. In consequence, both the composites derived from the parent CTA-Mt and the ones obtained from the Na-Mt component were compositionally similar. However, preparation of Na-Mt in microemulsion led to better disintegration of clay particles than the exfoliation treatment of CTA-Mt in an organic solvent. This resulted in the TiO_2_/Na-Mt series showing superior intermixing of clay component with titania nanoparticles and ensured formation of composites with better-developed pore networks while retaining similar pore size distribution characteristics. The advantage of the adopted synthesis method is the formation of essentially mesoporous materials, with significant specific surface area and uniform mesoporosity determined by the size of titania nanoparticles. Given that the size of the inverse micelles can be modified by appropriate selection of the synthesis conditions, this method offers the possibility to control the dimensions of the nanoparticles formed in the micellar cores. This aspect of TiO_2_/clay mineral synthesis as well as the photocatalytic properties of composites synthesized according to the procedure described in this paper are the matter of our current work.

## Figures and Tables

**Table 1:** Chemical composition of investigated materials (based on EDX analysis) and textural parameters from N_2_ adsorption/desorption isotherms at −196 °C.

Sample	TiO_2_ (wt. %)	SiO_2_ (wt. %)	Al_2_O_3_ (wt. %)	MgO (wt. %)	Fe_2_O_3_ (wt. %)	Na_2_O (wt. %)	S_BET_ (m^2^g^−1^)	V_tot_ (cm^3^g^−1^)	V_meso_ (cm^3^g^−1^)	D_av_ (nm)	D_dom_ (nm)
0.25TiO_2_/CTA-Mt	16.4	56.3	21.6	3.8	1.9	-	174	0.233	0.174	5.3	3.8
0.5TiO_2_/CTA-Mt	35.1	43.6	16.5	2.8	2.0	-	184	0.217	0.160	4.7	4.6
1TiO_2_/CTA-Mt	45.2	36.5	14.4	2.2	1.7	-	208	0.247	0.189	4.8	4.9
1.5TiO_2_/CTA-Mt	59.7	27.0	10.2	1.9	1.2	-	194	0.246	0.208	5.1	5.2
0.25TiO_2_/Na-Mt	17.5	55.7	21.3	3.8	1.7	-	223	0.321	0.259	5.8	4.4
0.5TiO_2_/Na-Mt	37.4	42.4	16.0	2.7	1.5	-	245	0.313	0.248	5.1	4.9
1TiO_2_/Na-Mt	48.3	34.9	13.7	1.9	1.2	-	256	0.321	0.263	5.0	5.2
1.5TiO_2_/Na-Mt	63.9	24.4	9.4	1.4	0.9	-	231	0.314	0.274	5.5	5.7
calcined Na-Mt	-	65.6	24.9	4.5	2.1	2.9	47	0.129	0.111	11.1	4.6
calcined CTA-Mt	-	66.5	25.8	4.9	2.8	-	92	0.168	0.136	7.3	4.0
calcined TiO_2_	100.0	-	-	-	-	-	12	0.079	0.079	26.4	5.8

S_BET_, BET-specific surface area; V_tot_, total pore volume; V_meso_, BJH volume of mesopore; D_av_, average pore dimension; D_dom_, dominant pore size.

**Table 2:** Comparison of textural properties of TiO_2_/Mt composites obtained by various methods reported in the literature with those of the materials synthesized in this study.

Method of TiO_2_–Mt Composite Preparation	S_BET_ (m^2^g^−1^)	V_tot_ (cm^3^g^−1^)	Reference
Pillaring of Mt with TiCl_4_ precursor, calcination 400 °C	379	0.392	[35]
Pillaring of Mt with TiCl_4_ precursor, hydrothermal treatment 115 °C calcination 500 °C	135	0.303	[36]
Pillaring of Mt with Ti(OC_3_H_7_)_4_ precursor, hydrothermal treatment 60 °C, calcination 500 °C	183	0.19	[37]
Pillaring of Mt with Ti(OC_4_H_9_)_4_ precursor, calcination 450 °C	133	0.335	[38]
Pillaring of CTA-Mt with Ti(OC_3_H_7_)_4_ precursor, calcination 500 °C	194	0.165	[39]
Porous clay heterostructure synthesis with Ti(OC_3_H_7_)_4_ precursor and commercial organoclay Cloisite^®^30B, calcination 550 °C	143	0.210	[33]
Mt impregnated with TiCl_4_ precursor, calcination at 350 °C	52	0.144	[34]
TiCl_4_ precursor hydrolyzed within inverse micelles, mixed with exfoliated CTA-Mt, calcination 550 °C	208	0.247	this work
TiCl_4_ precursor hydrolyzed within inverse micelles, mixed with Na-Mt dispersed in inverse microemulsion, calcination 550 °C	256	0.321	this work

S_BET_, BET-specific surface area; V_tot_, total pore volume.

## Data Availability

The data presented in this study are available on request from the corresponding author.
